# Structural basis of direct and inverted DNA sequence repeat recognition by helix–turn–helix transcription factors

**DOI:** 10.1093/nar/gkac1024

**Published:** 2022-11-12

**Authors:** Raul Fernandez-Lopez, Raul Ruiz, Irene del Campo, Lorena Gonzalez-Montes, D Roeland Boer, Fernando de la Cruz, Gabriel Moncalian

**Affiliations:** Departamento de Biología Molecular, Universidad de Cantabria and Instituto de Biomedicina y Biotecnología de Cantabria (IBBTEC), CSIC-Universidad de Cantabria, 39011, Santander, Spain; Departamento de Biología Molecular, Universidad de Cantabria and Instituto de Biomedicina y Biotecnología de Cantabria (IBBTEC), CSIC-Universidad de Cantabria, 39011, Santander, Spain; Departamento de Biología Molecular, Universidad de Cantabria and Instituto de Biomedicina y Biotecnología de Cantabria (IBBTEC), CSIC-Universidad de Cantabria, 39011, Santander, Spain; Departamento de Biología Molecular, Universidad de Cantabria and Instituto de Biomedicina y Biotecnología de Cantabria (IBBTEC), CSIC-Universidad de Cantabria, 39011, Santander, Spain; Alba Synchrotron, Cerdanyola del Vallès, 08290, Barcelona, Spain; Departamento de Biología Molecular, Universidad de Cantabria and Instituto de Biomedicina y Biotecnología de Cantabria (IBBTEC), CSIC-Universidad de Cantabria, 39011, Santander, Spain; Departamento de Biología Molecular, Universidad de Cantabria and Instituto de Biomedicina y Biotecnología de Cantabria (IBBTEC), CSIC-Universidad de Cantabria, 39011, Santander, Spain

## Abstract

Some transcription factors bind DNA motifs containing direct or inverted sequence repeats. Preference for each of these DNA topologies is dictated by structural constraints. Most prokaryotic regulators form symmetric oligomers, which require operators with a dyad structure. Binding to direct repeats requires breaking the internal symmetry, a property restricted to a few regulators, most of them from the AraC family. The KorA family of transcriptional repressors, involved in plasmid propagation and stability, includes members that form symmetric dimers and recognize inverted repeats. Our structural analyses show that ArdK, a member of this family, can form a symmetric dimer similar to that observed for KorA, yet it binds direct sequence repeats as a non-symmetric dimer. This is possible by the 180° rotation of one of the helix–turn–helix domains. We then probed and confirmed that ArdK shows affinity for an inverted repeat, which, surprisingly, is also recognized by a non-symmetrical dimer. Our results indicate that structural flexibility at different positions in the dimerization interface constrains transcription factors to bind DNA sequences with one of these two alternative DNA topologies.

## INTRODUCTION

Key cellular processes such as transcriptional regulation and DNA repair depend on the ability of proteins to bind certain sites in the genome. A handful of structural motifs confer DNA-binding proteins (DBPs) their ability to recognize specific sequences. The specificity of DBPs depends on two general mechanisms (1). On the one hand, DBPs exhibit a ‘base readout’ mechanism, in which specific contacts between DNA bases and amino acid side chains on the protein determine the binding affinity. On the other hand, DBP binding also relies on the overall structure of the protein and its cognate site. Shape readout mechanisms depend on the global architecture of the complex, thus their dependency on DNA and protein sequences is subtler, and has proved more complicated to elucidate (2). At the DNA-binding site, curvature and flexibility have been shown to be key determinants of binding specificity (3). At the protein level, there is evidence that structural constraints located outside the DNA-binding region, such as the presence of intrinsically disordered regions (4), or allosteric interactions in the dimerization domains (5), may also play a key role. Overall, despite substantial advances in our understanding of the structural basis of DBP specificity, predicting binding sites and designing new DBPs with engineered specificity remain challenging tasks (6–8).

Among the different DNA-binding domains, the helix–turn–helix (HTH) is one of the best studied and most ubiquitous (9). In bacteria, transcription factors (TFs) containing HTH domains typically recognize operators with an inverted repeat (IR) architecture. Classical examples of HTH-containing TFs recognizing palindromic sequences include the cI, TetR and LacI families (10). Base readout mechanisms of HTH motifs typically involve contacts with three base pairs in the major groove of the DNA (11,12) although sequence-specific DNA binding could also be determined by contacts outside the HTH motif (13). Such a short contact interface requires several HTH motifs acting in a concerted fashion in order to achieve stable binding (9). In some cases, a single protein contains several HTH motifs and binds DNA as a monomer, such as, for example, prokaryotic sigma factors. However, the most common arrangement for bacterial TFs is the formation of protein oligomers, where contacts with the DNA are formed by HTHs motifs of different subunits. These oligomers typically adopt a head-to-head configuration, which in turn selects for IR operator architectures. Binding of direct sequence repeats (DRs) requires the protein adopting a head-to-tail configuration. Any globular protein adopting such an arrangement is susceptible to the uncontrolled formation of trimers, tetramers and higher order oligomers. This structural constraint is probably the reason why IRs are more abundant than DRs in TF operators (9). This does not entirely preclude, however, DR binding by HTH-containing TFs. Members of the AraC family recognize DRs while forming symmetric dimers (14). This is achieved by the structural independence of the dimerization and DNA-binding domains, which in AraC-like TFs have an independent globular structure connected by a flexible linker. This structural independence allows the dimerization interface to adopt a head-to-head arrangement, while the DNA-binding domains keep a head-to-tail configuration. HTH-containing TFs belong to families that show a preference for either IRs or DRs, depending on their structural constraints. There are some indications, however, that this preference may not be intrinsic to the overall fold of the protein. Some members of the TetR family, such as ComA, are able to bind IRs and DRs, yet the molecular mechanisms behind this ability are unknown (15).

The KorA family of transcriptional regulators includes plasmid-borne repressors involved in plasmid stability and conjugation (16–18). The canonical representative of this TF family, KorA from broad host range plasmid RP4, forms a symmetric dimer with two tri-helical HTH domains. KorA recognizes and binds a conserved IR located at the –10 region of its target promoters, a feature presumably shared by other members of the family (19,20). A second group of KorA homologues (36% identical at the amino acid level) is represented by ArdK proteins from IncN and IncW plasmids (21,22). ArdK TFs do not display IRs in their target promoters. Instead, they show conserved DRs that were proposed as their cognate operators (21–23). In an effort to elucidate the structural bases for IR and DR recognition in these homologous TFs, we obtained the *a*po and holo structures of ArdK. Structural and experimental analyses showed that, while the overall conservation between KorA and ArdK proteins remains high, minor changes at the dimerization domain modify the structural flexibility of these proteins. This, in turn, alters the shape readout mechanism of the TF, switching its preference from a DR structure to an IR structure. Our results illustrate how subtle structural constraints hidden in regions not directly involved in DNA recognition may dramatically alter the specificity of DNA-binding proteins.

## MATERIALS AND METHODS

### Strains and culture conditions

*Escherichia coli* DH5α strain was employed for cloning procedures and *E. coli* strain C41 (DE3) for protein expression. Expression profiling was performed in *E. coli* Bw27783v, a strain that constitutively expresses the *araE* transporter and allows regulatable pBAD induction (24). Liquid cultures were prepared in flasks containing 1/4 volume LB medium (Pronadisa, Spain) supplemented with kanamycin sulphate (Sigma Aldrich) at a final concentration of 50 μg/ml and incubated with shaking (180 rpm). For solid media culture, LA was used [LB medium supplemented with 1.5% (w/v) agar].

### DNA manipulation and plasmid construction

Plasmids with promoters containing DRs or IRs with both arms separated by a different number of bases (Supplementary Table S2) were constructed using pGP8 (*Pssb:GFP*) as template. IR_DIR and the different IR or DR primers shown in Supplementary Table S1 were used to generate the desired plasmids by polymerase chain reaction (PCR). PCR products were then treated with DpnI and transformed into competent *E. coli* DH5α cells.

R388 plasmid was used as template to clone *ardK* into pET29c to give rise to plasmid pARA (Supplementary Table S2) by the isothermal assembly Gibson method (25). DNA fragments were amplified by PCR with oligonucleotides purchased from Sigma-Genosys (Sigma-Aldrich) and the DNA polymerase Phusion (Thermo fisher, EEUU). PCR products were extracted from agarose gels by using the GenElute Gel Extraction kit (Sigma-Aldrich) and its concentration determined with a Nano-Drop ND-1000 spectrophotometer (Thermo Scientific). Isothermal assembly reactions were transformed into competent *E. coli* DH5α cells by electroporation using 0.2 cm Gene Pulser cuvettes (BioRad) in a MicroPulser™ electroporator (BioRad). The polymerase Biotaq (Bioline) was used for PCR verification of the genetic constructions.

### Primer extension

Total RNAs were prepared from *E. coli* cells harbouring pUA66-derived plasmids (Supplementary Table S2) grown at 37°C until OD_600_ = 0.5–0.7. Harvested cells were treated with RNAprotect Bacteria Reagent (Qiagen) and snap-frozen. Cells were lysed with lysozyme (Sigma-Aldrich) and proteinase K (Roche). Total RNA was extracted with the RNeasy Mini Kit (Qiagen) and treated with RNase-free DNase (Qiagen) in column for DNA removal. Ambion TURBO DNA-free DNase treatment was also applied for better DNA removal. RNA integrity and quality were validated by the Agilent RNA ScreenTape assay. The RNA integrity number equivalent (RINe) was assured to be >8 to use the isolated RNA in the RNA-seq experiment (26).

Green fluorescent protein (GFP)-seq primer was used for the identification of the transcription start site of the promoters of the pUA66-derived plasmids. GFP-seq (GGGACAACACCAGTG) anneals within the pUA66 *gfp* gene, 24 bp downstream of the GFP start codon. GFP-seq was radiolabelled at its 5′ end using ^32^P. GFP-seq (56 pmol) was mixed with 3 μl of [γ-^32^P]ATP (10 mCi/ml) and 10 U of T4 polynucleotide kinase (Amersham) to a final volume of 50 μl in the T4 polynucleotide kinase buffer. After 30 min at 37°C, the enzyme was inactivated at 90°C for 10 min, and radiolabelled GFP-seq was purified using a Microspin G-25 column (Amersham).

For the primer extension reaction, radiolabelled GFP-seq oligonucleotide was annealed to RNA (5 μg) at 65°C, 5 min. Then, a mixture of dNTP (100 mM each) was added. The reactions were started by the addition of 8 U of AMV reverse transcriptase (Promega) and left to proceed for 30 min at 42°C. Reactions were ethanol precipitated, and dissolved in 8 μl of loading solution (95% formamide, 20 mM EDTA, 0.05% bromophenol blue, 0.05% xylene cyanol). Samples were then loaded on 8% sequencing gels and electrophoresed at 1800 V. Sequencing reactions performed using the fmol DNA Cycle Sequencing system (Promega) were run as controls to measure the size of the extended oligonucleotide. The ^32^P-labelled bands were detected using the Molecular Imager FX system (Biorad).

### Expression profiling

pAR4 plasmid was transformed into *E. coli* Bw27783 containing the corresponding reporter plasmids (Supplementary Table S2) as described in (23). Protein expression was induced by adding appropriate concentrations of arabinose to M9-broth and promoter expression levels were determined following the protocol detailed in (27). Briefly, cells were pre-grown for 16 h in the presence of the appropriate arabinose concentrations. Cultures were then diluted 1:10 000 in the same medium and grown for 6 h to ensure that measurements were made in pseudo steady state. Fluorescence per OD unit (GFP/OD) was measured and averaged around steady-state levels. Steady-state values obtained when inducing ArdK with different arabinose concentrations were compared with those produced by the same reporter strain when it contained the empty expression vector pBAD33.

### Phylogenetic analysis

Members of the KorA/ArdK family were retrieved from the databases by BLAST using as baits the amino acid sequences of KorA from plasmid RP4 and ArdK from plasmid R388. Proteins were aligned using ClustalW and, from this alignment, Neighbor–Joining and maximum likelihood trees were built. Bootstrapping values were obtained with at least 1000 iterations.

### Protein expression and purification

ArdK-derived proteins containing a C-terminal His tag (ArdKHT) were purified as follows. An overnight culture of *E. coli* BL21 (DE3) cells harbouring pARA (pET29c::*ardK*) plasmid was diluted 20-fold in 1 litre of 2× yeast extract–tryptone (YT) medium containing kanamycin and incubated at 37°C with shaking until *A*_550_ = 0.6. Then, isopropyl-β-d-thiogalactopyranoside (IPTG) was added to a final concentration of 0.5 mM. After 3 h further incubation, cells were pelletted and resuspended in 50 ml of buffer A (100 mM Tris–HCl, 500 mM NaCl pH 7.0). After cell lysis by sonication, ArdKHT-containing supernatant was loaded onto a 5 ml HisTrap column (Amersham) and eluted with a linear imidazole gradient (0–500 mM). ArdKHT-containing fractions were pooled, concentrated to 2 ml and loaded onto a HiLoad 16/60 Superdex 200 gel filtration column equilibrated with buffer B [20 mM Tris (pH 7.5), 150 mM NaCl, 1 mM dithiothreitol (DTT), 1 mM EDTA].

ArdKHT labelled with seleno-methionine (SeMet) was obtained using the B834(DE3) strain and minimal medium supplemented with SeMet (28). The purification was performed according to the described procedure.

### Complex formation, crystallization, X-ray data collection and processing

Crystals of apo ArdK-SeMet were obtained using the sitting-drop vapour diffusion method at 22°C by mixing 2 μl of protein at 9 mg/ml concentration in 20 mM Tris–HCl, 150 mM NaCl, 1 mM EDTA buffer with 1 μl of the reservoir solution containing 2 M sodium formate. Data were collected at 0.9794 Å, the wavelength corresponding to the selenium absorption maximum according to the fluorescence scan at 105 K from a crystal transferred to cryoprotectant solution B [20% (v/v) ethylene glycol, 1.6 M sodium formate].

Datasets were obtained at beamline BM16 at the ESRF European Synchrotron Radiation Facility (Grenoble, France).

For the structural analysis of ArdK bound to DR, ArdK (at 5 mg/ml in 20 mM Tris–HCl, 150 mM NaCl, 1 mM EDTA buffer) and DR double-stranded DNA (dsDNA) substrate (5′–3′) were mixed at a 1:2 protein:DNA molar ratio. After 30 min incubation at 22°C, the ArdK–DR complex was concentrated up to 10 mg/ml using an Amicon Ultra-15 10K device (10,000 MWCO). Crystals were grown with sitting-drop vapour diffusion at 22°C by mixing 2 μl of ArdK–DR complex with 1 μl of reservoir solution containing 20% polyethylene glycol (PEG) 4000, 10% 2-propanol and 0.1 M Tris–HCl (pH 7.5). Data were collected at 0.9790 Å, the wavelength corresponding to the selenium absorption maximum according to the fluorescence scan at 105 K from a crystal transferred to cryoprotectant solution C [20% (v/v) ethylene glycol, 16% PEG 4000, 8% 2-propanol and 80 mM Tris–HCl (pH 7.5)].

Datasets were obtained at beamline PROXIMA at the SOLEIL Synchrotron Radiation Facility (Paris, France).

Crystals of ArdK bound to IR3 were obtained by mixing ArdK [at 5 mg/ml in 20 mM Tris–HCl, 150 mM NaCl, 1 mM EDTA buffer] and IR3 dsDNA substrate (5′–3′) at a 1:2 molar ratio. After 30 min incubation at 22°C, the ArdK–IR3 complex was concentrated up to 10 mg/ml using a Amicon® Ultra-15 10K device (10,000 MWCO). Crystals were grown with sitting-drop vapour diffusion at 22°C by mixing of 2 μl ArdK–IR complex with 1 μl of reservoir solution containing 20% PEG 8000 and 0.1 M Tris–HCl (pH 8.5). Data were collected at 0.9792 Å and 105 K from a crystal transferred to cryoprotectant solution D [20% (v/v) ethylene glycol, 16% PEG 8000 and 80 mM Tris–HCl (pH 8.5)].

Datasets were obtained at beamline XALOC at the ALBA Synchrotron Radiation Facility (Barcelona, Spain).

Diffraction images were processed using iMosflm and Scala as part of the CCP4 package (29). The structures of apo ArdK-SeMet and ArdK-SeMet–DR were solved by single anomalous dispersion (SAD) phasing using the program AutoSol of the PHENIX package (30). The ArdK–IR3 structure was solved by molecular replacement using the program Phaser-MR of the PHENIX package (30) and the ArdK-SeMet–DR structure as a search model. The refinement of the initial models was performed through several cycles by Phenix refine (30) until appropriate R factors were reached. Final manual modelling was done in COOT (31).

## RESULTS

### ArdK represses the expression of five genes in the R388 plasmid, all of them containing a conserved DR within their promoter regions

**Figure 1.**
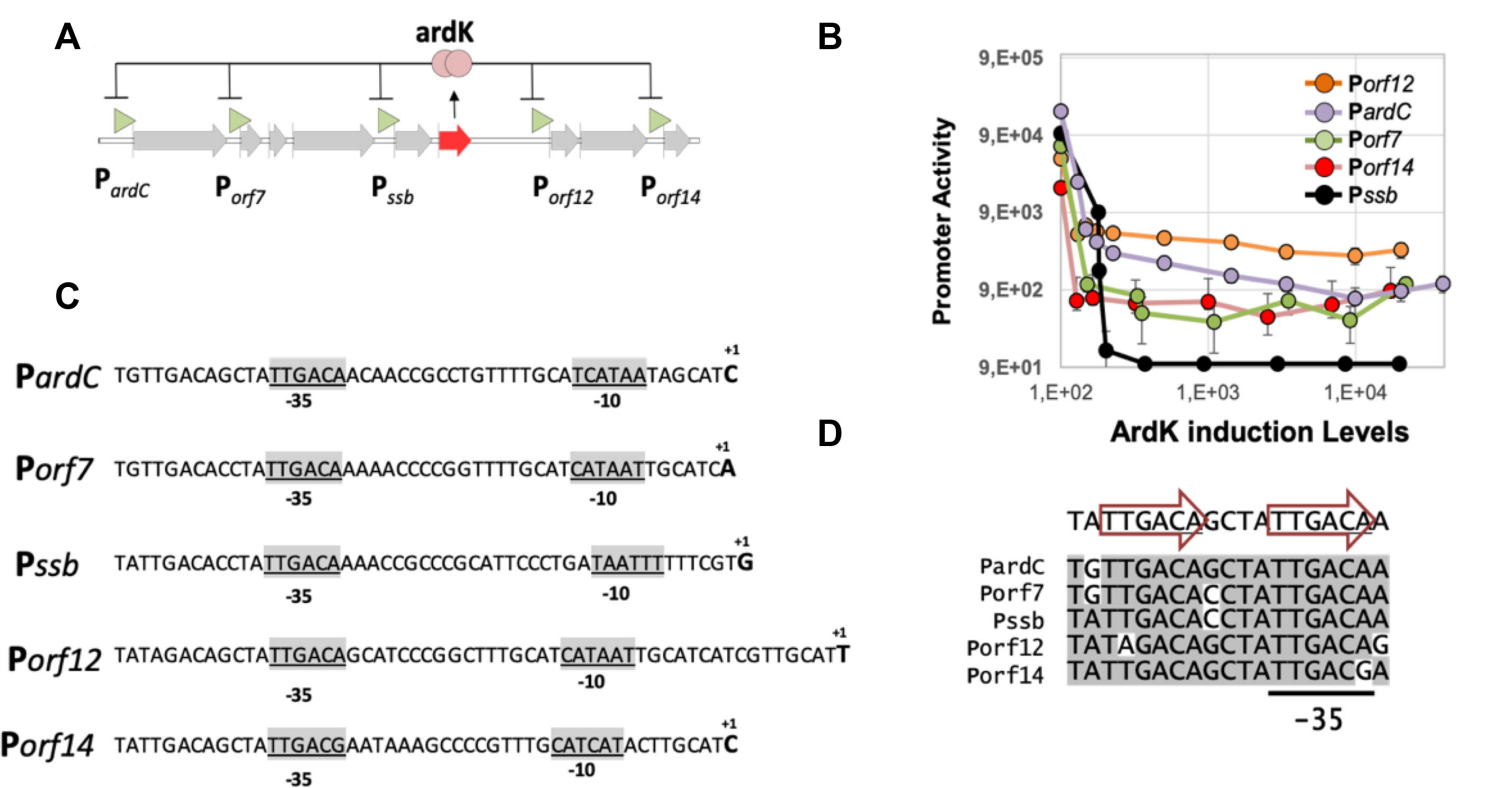
Circuit, repression and binding sites of ArdK. (**A**) The regulatory circuit of ArdK in plasmid R388. In red, *the ardK* gene and protein. In green, target promoters for ArdK in the plasmid R388 genome. (**B**) Transcriptional activity of ArdK target promoters (*y*-axis) in response to different levels of ArdK induction (*x*-axis). Dots and error bars represent, respectively, the average and standard deviation (SD) of four independent experiments. Measurements without visible error bars correspond to those with an SD so small that dots and error bars overlap. (**C**) Location of the transcriptional start sites (+1) and the putative –10 and –35 boxes within the five promoters regulated by ArdK (**D**) Sequence conservation in ArdK target promoters. Conserved bases are highlighted in grey. Arrows indicate the location of two direct sequence repeats.

ArdK is a 102 amino acid protein encoded by the broad host range conjugative plasmid R388. In R388, ArdK represses the expression of five genes by its binding to their respective promoters (P*ardC*, P*orf7*, P*ssb*, P*orf12* and P*orf14*, shown in Figure 1A) (23). As in its close homologue, ArdK from plasmid pKM101, these promoters are involved in the early steps of invasion of a new bacterial host after the plasmid is transferred by conjugation (22). Using expression profiling, we tested the response of these promoters to increasing levels of ArdK. For this purpose, ArdK was cloned under a regulatable pBAD promoter, which responds to the presence of arabinose (24). This construction was introduced into *E. coli* BW27783 cells harbouring transcriptional fusions of ArdK target promoters to the GFP gene, as described in the Materials and Methods. ArdK was induced at different levels, and gene expression from its target promoters was monitored by measuring GFP fluorescence. The results, shown in Figure 1B, indicated that all target promoters sharply reduced *gfp* expression, although the level of repression was variable. Some promoters showed a three orders of magnitude decrease (pSsb), while others decreased only 15-fold (pORF12) at saturating ArdK concentrations. We employed primer extension analysis to determine the transcriptional start sites, and the putative location of –10 and –35 boxes (Figure 1C; Supplementary Figure S1). Sequence alignment of ArdK target promoters revealed the presence of a conserved DNA motif, likely to contain ArdK operator, situated adjacent to the –35 box of target promoters (Figure 1D). The sequence TTGACA is perfectly repeated in all the promoters except in the least repressed promoter P*orf12*.

### ArdK overall structure

The possibility of binding to DRs located next to the –35 box of target promoters was in sharp contrast to data available for KorA, the canonical representative of this TF family. KorA_RP4 is 36% identical at the amino acid level to ArdK_R388, yet structural and molecular data indicate that KorA binds to an IR located at the –10 box (19,20). To compare both proteins, we purified ArdK as a His-tag protein fusion and obtained its structure by X-ray crystallography. ArdK was crystallized as described in the Materials and Methods, and resolved to 3.0 Å resolution (Table 1).

**Table 1.** Data collection and refinement statistics

	ArdKSE	ArdKSE–DR	ArdK–IR3
**Wavelength (Å)**	0.9794	0.9790	0.9792
**Resolution range (Å)**	63.63–3.0 (3.16–3.0)	58.78–2.8 (2.95–2.8)	77.22–2.6 (2.74–2.6)
**Space group**	P 41 21 2	C 1 2 1	P 21 21 21
**Unit cell**	43.1 43.1 190.89 90 90 90	88.12 39.06 120.04 90 101.65 90	43.89 77.22 114.12 90 90 90
**Total reflections**	26 971 (3904)	34 348 (5141)	56 402 (8220)
**Unique reflections**	4103 (565)	10 025 (1437)	12 277 (1744)
**Multiplicity**	6.6 (6.9)	3.4 (3.6)	4.6 (2.6)
**Completeness (%)**	99.8 (100)	98.9 (99.9)	98.3 (98.4)
**Mean I/sigma(I)**	11.3 (3.8)	10.4 (2.7)	8.1 (4.7)
**Wilson B-factor**	95.9	77.2	60.8
**R-merge**	0.097 (0.371)	0.051 (0.335)	0.084 (0.362)
**R-meas**	0.112 (0.428)	0.071 (0.462)	0.102 (0.446)
**CC1/2**	0.992 (0.963)	0.997 (0.901)	0.995 (0.940)
**Reflections used in refinement**	4047 (399)	9871 (956)	9750 (1175)
**Reflections used for R-free**	221 (25)	490 (50)	465 (39)
**R-work**	0.2462 (0.3813)	0.2317 (0.3720)	0.2538 (0.4063)
**R-free**	0.2686 (0.5738)	0.2894 (0.5041)	0.2988 (0.4320)
**CC(work)**	0.844 (0.665)	0.880 (0.705)	0.690 (0.740)
**CC(free)**	0.840 (0.390)	0.880 (0.247)	0.842 (0.632)
**Number of non-hydrogen atoms**	735	2268	2232
**Protein residues**	94	189	191
**RMS(bonds) (Å)**	0.013	0.004	0.006
**RMS(angles) (°)**	1.46	0.72	0.78
**Ramachandran favoured (%)**	95.65	95.14	94.65
**Ramachandran allowed (%)**	4.35	4.86	5.35
**Ramachandran outliers (%)**	0.0	0.0	0.0
**Rotamer outliers (%)**	3.38	2.48	3.75
**Clashscore**	3.38	10.42	13.91
**Average B-factor (Å^2^)**	86.77	86.40	66.08

Statistics for the highest-resolution shell are shown in parentheses. Refinement statistics were calculated using PHENIX (30).

**Figure 2.**
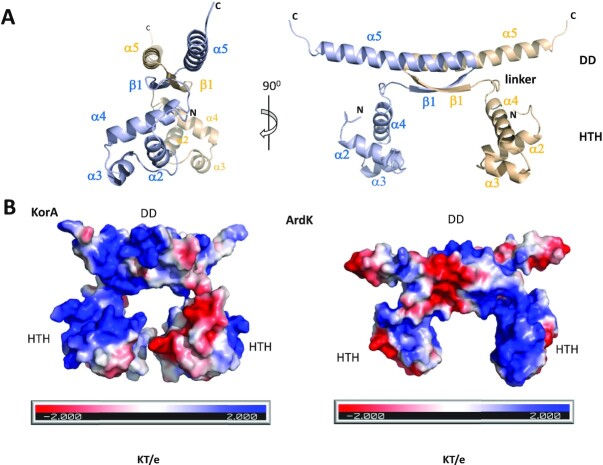
ArdK structure. (**A**) Orthogonal views of the structure of ArdK. The DD and HTH domains, the linker region and the secondary structure elements of ArdK are shown. One of the subunits is coloured in beige and the other in grey. (**B**) Comparison between ArdK and KorA (pdb 2N5G) solvent-accessible surface electrostatic potential. Negative potential is coloured in red and positive potential in blue from –2 kT/e to + 2 kT/e (calculated by APBS).

In the crystal of the apo structure, there is a single ArdK molecule in the asymmetric unit. This molecule contains an N-terminal HTH domain (residues 1–62) and a C-terminal domain with a C-terminal α-helix (residues 75–102) connected by a linker region (residues 63–74). The C-terminal domain forms a β-sheet with its symmetry-related molecule. Thus, the biological assembly (Figure 2A) is very probably a dimer containing two HTH domains (α1–α4) and a dimerization domain (DD, β1 and α5). In fact, according to S75 gel filtration data, the protein purified as a dimer in solution.

The structure of the HTH and DD domains in ArdK is strikingly similar to the structure of these domains in KorA (19,20). Both the HTH and the DD of ArdK show a root mean square deviation (RMSD) of 0.83 Å and 1.35 Å with their respective homologue domains in KorA (Supplementary Figure S2). The only significant difference was the relative rotation of the HTH domain with respect to the DD. Comparison between both structures revealed that both dimers are symmetrical, but the orientation of the HTH differs: in ArdK, the HTH domains are rotated 150° with respect to their position in KorA (Supplementary Figure S3). As a consequence, the electropositive N-terminal ends of the recognition α4 helices point towards each other in KorA, whereas they are pointing in opposite directions in ArdK (Figure 2B).

### Crystal structure of the ArdK–DR DNA complex

Binding of KorA to its cognate operator requires a rotation of 45° of both HTH domains, to allow the DNA to be inserted between them (Supplementary videos 1 and 2). KorA straddles on its target DNA, aided by a flexible linker between the HTH and DD domains (20). In ArdK, the rotation of the HTH domains relative to the DD made such movement unlikely. To study the binding mechanism of ArdK, we obtained the crystal structure of the protein bound to its cognate DNA operator. We chose the operator present in the P*ssb* promoter (GTATTGACACCTATTGACA; underlining indicates the –35 box), as this was the promoter with the highest level of repression by ArdK (Figure 1B). Two complementary 19mer oligonucleotides containing this sequence were annealed and bound to SeMet ArdK, as described in the Materials and Methods. Crystals of the ArdK–DNA complex were obtained, and the structure solved at 2.8 Å resolution (Table 1).

**Figure 3.**
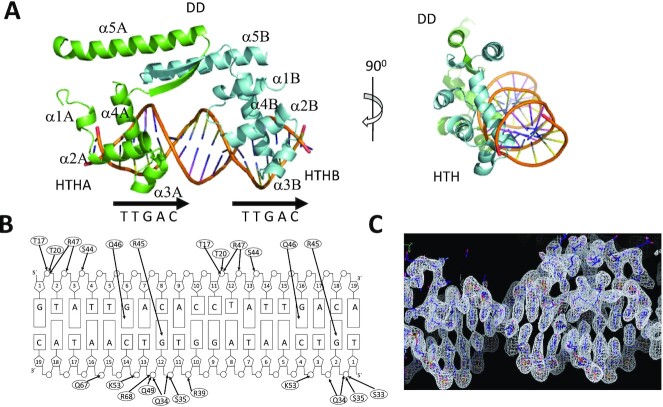
(**A**) Orthogonal views of the crystal structure of ArdK bound to DR DNA. One of the subunits of ArdK is coloured in green and the other in cyan. The DD and HTH domains and the secondary structure elements of ArdK are labeled. Location of the DNA direct repeats is shown by arrows. (**B**) Recognition of specific bases in the operator. Hydrogen bonds between ArdK and the DNA are shown by black arrows. (**C**) 2Fo–Fc electron density map of the ArdK HTHB DNA interaction region.

In the ArdKSE–DR crystal structure, we found an ArdK dimer bound to a single dsDNA molecule within the asymmetric unit. Each of the DR repeats is bound by each of the ArdK HTH domains (Figure 3). The main residues involved in specific interactions with the recognized sequence were R45 and Q46. R45 NH2 interacted with G2_C or G12_C O6, R45 Nϵ with G2_C or G12_C N7, and Q46 Nϵ^2^ with G6_D or G16_D O6. Hydrogen bonds with the DNA backbone phosphates, and van der Waals forces stabilize the DNA interaction (Figure 3B). To achieve this rather unusual binding architecture, one of the HTHs had to rotate 180° relative to its apo position, while the other HTH motif and the dimerization domain remained at their original position (Supplementary Figure S4; Supplementary videos 3 and 4). The partial overlap of the –35 box is reflected in the structures involved in its recognition, since the recognition motif of ArdK is identical to the HTH of sigma 70 protein contacting its –35 box (32) (Supplementary Figure S5). The DALI server showed that, after KorA, the closest structural homologue to the ArdK HTH domain was indeed an RNA polymerase sigma factor (PDB 1KU7, RMSD 1.52 Å).

**Figure 4.**
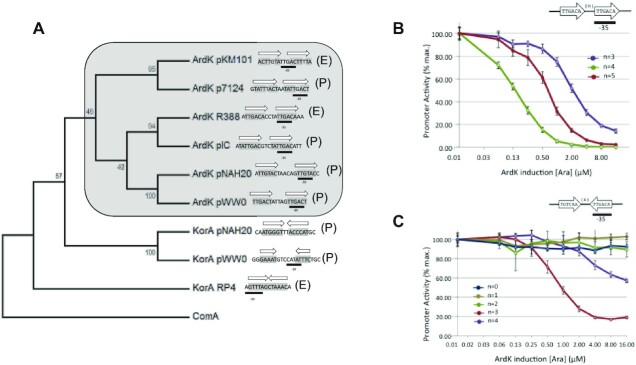
(**A**) Evolution of the KorA/ArdK family. Phylogenetic tree of ArdK/KorA family members showing their experimental (E) or putative (P) operators. The tree was built using the Neighbor–Joining algorithm, and numbers on the branches indicate bootstrap values. (**B**) ArdK is able to repress DRs with different spacers. Transcriptional activity of a synthetic promoter containing a TTGACA DR, with both arms separated by the number of bases indicated in the key. Promoter activity, expressed as the percentage of the maximum GFP/OD_600_ value (*y*-axis), is plotted against the ArdK induction level (*x*-axis). Dots and error bars represent, respectively, the average and SD of three independent experiments. (**C**) ArdK is able to repress IRs. Transcriptional activity of a synthetic promoter containing a TTGACA IR, separated by a loop of variable length, as shown in the key. Promoter activities and induction levels are expressed as above.

Structural data thus indicated that ArdK recognizes a DR by dynamically breaking the dimer symmetry upon DNA binding. In KorA, flexibility at the hinge region of the dimerization domain allows the straddling of the palindromic operator. In contrast, in ArdK, it is the rotation of one of the HTH domains that allows the protein to adopt a DR recognition pattern (Figure 3;Supplementary Figure S4), straddling its operator DNA. It thus seems that, despite their high overall structural similarity, ArdK and KorA entail entirely different DNA recognition mechanisms. In an effort to elucidate the distribution of these two DNA binding modes within the KorA family, we retrieved homologues by BLAST search, and a phylogenetic tree of members of the KorA family was obtained, as described in the Materials and Methods (Supplementary Figure S6). An annotated version of the tree is shown in Figure 4A, in which we include only the KorA promoters in which the presence of IRs or DRs was found. In Figure 4A, these are marked as (E), when there is experimental evidence for the operator, and (P) when the operator is putative and based on DNA sequence inspection alone. As shown in the figure, ArdK proteins form a monophyletic branch exhibiting DRs always in the close vicinity of the –35 box. In contrast, KorA homologues with IRs populate other branches of the tree. Although proteins from the family can be classified according to operator topology (DR/IR), there is substantial variation between operators of the same topological structure. For example, DRs from the ArdK subfamily present little sequence conservation, besides partial overlap with the sigma70 –35 box. They also differ in the relative position of the –35 box with respect to the arms of the direct repeat (between repeats in pKM101 or as part of one of the repeats in R388). The spacing between repeats is also variable, from 3 bp in pKM101 to 6 bp in pNAH20. This variation is also found in the KorA subfamily: KorA from plasmid RP4 binds an IR with no separation between each of the symmetric arms, but the IR found for KorA_pWW0 presents a 6 bp spacer. Altogether, the data suggest a remarkable flexibility in this TF family, enabling the members to bind DR and IR operators with different arm separations.

### ArdK–IR DNA binding

The variety of DNA configurations recognizable by KorA/ArdK homologues, along with the structural flexibility observed in ArdK and KorA, posed the question of whether the same protein was able to bind both IRs and DRs. To this end, we tested the ability of a pBAD::ardK expression vector to repress transcription from target promoters containing different topological structures. First, we determined whether ArdK could repress promoters containing DR operators with different spacers between the arms. To this end, we built three synthetic promoters containing two perfect ArdK recognition arms (TTGACA) in a DR conformation, separated by 3, 4 and 5 bp, respectively (see the Materials and Methods). As shown in Figure 4B, ArdK repressed the transcriptional activity of all three. Total repression was achieved at lower ArdK levels for the wild-type 4 bp spacer, while 3 bp and 5 bp spacers required higher ArdK doses to shut down transcriptional activity. The curves were displaced to higher ArdK concentrations, indicating an increase in the apparent binding constant *K*, but the slope of the curve did not change, indicative of unmodified cooperativity. We then analysed whether ArdK retained the ability to repress operators with an IR configuration, similar to the topology recognized by KorA. To test this hypothesis, we inverted the proximal arm of the DR, forming an IR with a variable separation between arms (Figure 4C). We tested five different spacer lengths, from *n* = 0 to *n* = 4. As shown in the figure, when the spacer between IR arms was 3 or 4 bp, ArdK recognized and repressed the expression of the target promoter. Expression profiling indicated that the optimal distance was *n* = 3, a spacer distance that required ArdK concentrations similar to the DR with an *n* = 5 bp spacer.

**Figure 5.**
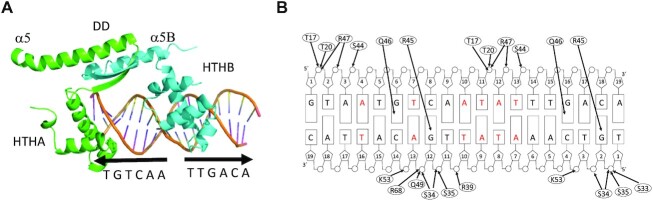
(**A**) Crystal structure of ArdK bound to IR DNA. One of the subunits of ArdK is coloured in green and the other in cyan. The DD and HTH domains of ArdK are shown. Location of the DNA inverted repeats is shown by arrows. (**B**) Recognition of specific bases in the operator. Hydrogen bonds between ArdK and the DNA are shown by black arrows. Bases different from those in the DR operator are shown in red.

ArdK was thus able to bind an IR, with *in vivo* efficiencies comparable with suboptimal DRs. To study the structural changes involved in this alternative binding mode, we obtained the crystal structure of ArdK bound to an IR with an *n* = 3 loop, as described in the Materials and Methods. The structure was solved at 2.6 Å resolution (Figure 5A). Strikingly, the IR–ArdK complex did not show any significant structural change compared with the DR–ArdK structure previously obtained. Despite the palindromic structure of the operator, the protein binds in a head-to-tail configuration. Furthermore, a close inspection of the DNA–protein contacts indicated that the DNA–protein interface was formed by the same amino acids (Figure 5B). The –35 box was again recognized by the rotating HTH motif, while the fixed HTH recognized the same GC base pair. The structure revealed the reason why ArdK was able to recognize that particular IR configuration: the *n* = 3 loop maintained the adventitious TGxCA repeat at the same distance as in its cognate DR architecture (Figure 5B). Bases located in between were different, but these were only contacted through their sugar backbone. Thus, although the operator had an apparent palindromic structure, it was contacted and recognized as a degenerate DR.

## DISCUSSION

Unravelling the molecular basis of TF specificity is key, not only to understand the complex regulatory networks that govern cell physiology, but also to be able to design new synthetic regulatory circuits. Position weight matrices, together with structural studies, have shed light on the specific determinants of base readout mechanisms (1). However, the sequence specificity of DBPs is often dependent on shape readout mechanisms, for which much less structural information is available (2). Here we have shown how minor changes in the dimerization domain of a classical HTH TF family radically alter the shape of the binding site recognized by the protein.

The KorA family of transcriptional regulators includes proteins that exhibit remarkable similarity in both functional and structural terms. Functionally, they are transcriptional repressors located in plasmids, controlling genes involved in ensuring plasmid propagation and stable maintenance (16–18). Engaged in negative feedback loops, these proteins experience a period of transitory overexpression after the plasmid is transferred into a new host by conjugation (23). Our results indicate that similarity among KorA members is also structural. The crystal structure of the apo form of ArdK from plasmid R388 is nearly identical to that of KorA from plasmid RP4. In both cases, the proteins form a symmetric dimer, with a tri-helical HTH domain for DNA binding, and an αβ dimerization interface. Despite this structural and functional similarity, the results showed that the mechanism for DNA binding was radically different. KorA uses a straddling mechanism to recognize a palindromic operator, located on the –10 box of its targets promoters (20). In contrast, the holo structure of ArdK revealed that this protein rotates one of its HTHs to break the internal symmetry and bind a DR located on the –35 box.

The phylogeny of this TF family revealed that ArdK homologues concentrate in a monophyletic branch of the tree (Figure 4A). Members of this ArdK-like subdivision exhibited conserved DRs overlapping the –35 boxes of their own promoters, suggesting that the rotational mechanism of binding is a conserved feature of these proteins. In contrast, other branches in the family included proteins that presented conserved IRs close to the –10 box, as in canonical KorA from plasmid RP4. Interestingly, we observed that IncP-9 plasmids from the *Pseudomonadaceae* contain both kinds of homologues in their genomes. In these plasmids, ArdK-like proteins presented –35 box DRs while KorA homologues showed –10 box IRs, as expected. This suggests that these subfamilies arose from duplication and functional divergence. Whether the ancestral protein was a KorA-like IR-binding or an ArdK-like DR-binding protein is not entirely clear due to the poor bootstrap resolution of some branches in the tree (Supplementary Figure S6). The monophyletic nature of ArdK-like proteins suggests, however, that these were most probably the proteins that emerged by duplication and divergence from a KorA-like ancestor.

Proteins that emerge by duplication and functional divergence often retain affinity for their former substrates. *In vivo* results with ArdK suggested that this was indeed the case, since the TF was able to repress IR-containing promoters. However, this was only possible when the IR arms were separated by at least 3 bp. This suggested that the mechanism of binding was not the same as in KorA, where both IR arms are adjacent to accommodate straddling of the protein. The IR-bound structure of ArdK confirmed this. ArdK is able to recognize an IR, but it binds in a head-to-tail configuration identical to that of the DR. The distance between the IR arms was necessary to situate the bases specifically recognized by each HTH domain (a GC pair) at the same distance as in the DR configuration. The rest of the DNA–protein interactions involve the sugar backbone, and thus are more tolerant to changes in the sequence. The results thus demonstrate that a TF may recognize operators with a DR or IR apparent topology without changes in the DNA binding mechanism. In the case of ArdK, binding to the non-preferred topology resulted in an apparent *in vivo* reduction of the affinity, as demonstrated by the repression index (<10-fold, compared with three orders of magnitude in the DR). However, variable affinity for different operator configurations is often found in TFs able to recognize both IRs and DRs, suggesting that this flexibility in DNA recognition may be more widespread than anticipated.

Altogether, the data also indicated that the DNA binding mechanism of a given TF may radically change with subtle changes in structural domains outside the DNA recognition region. In the case of Ardk and KorA, it is the flexibility of different regions of the dimerization domain that directs the protein towards straddling or domain rotation. Sequence alignments point to a conserved glycine in the Pro–Trp turn present in KorA and KorA-like proteins (Supplementary Figure S7A). In KorA, this Gly70 and the adjacent residue Glu69 freely rotate upon DNA binding (Supplementary Figure S7B; Supplementary video 5). In ArdK and ArdK-like proteins, this glycine is not conserved, and the equivalent residue is a glutamic acid (Glu65). Upon binding, there is no rotation of this amino acid (Supplementary Figure S7C; Supplementary video 6). As judged from the conserved sequences found in the target promoters of members of this family (Figure 4A), these subtle changes enable these TFs to recognize DRs and IRs with different spacing. Since alterations in the flexibility of unorganized regions of a protein are difficult to identify, it is possible that this structural flexibility is not exclusive to KorA/ArdK proteins, but common to other TFs. For example, changes at the dimerization interface of the glucocorticoid receptor, located outside the DNA-binding domain, determine sequence specificity (5). These results underline the importance of looking outside the DNA-binding domain to identify the structural constraints that direct DNA binding specificity. Moreover, they caution against assuming that the binding mechanism of a given TF may be directly inferred from sequence conservation. Instead, they underscore the need for detailed biophysical analyses to unravel the mechanisms behind the specificity of DNA-binding proteins.

## DATA AVAILABILITY

The atomic structures determined in this work have been deposited in the Protein Data Bank (PDB) under accession codes 7BBQ (apo), 7BCA (DR-bound) and 7BCB (IR-bound).

## Supplementary Material

gkac1024_Supplemental_Files
