# Supplementary material for: Structural basis of direct and inverted DNA sequence repeat recognition by helix–turn–helix transcription factors
Source: Nucleic Acids Res. 2022 Nov 12;50(20):11938–47. doi: 10.1093/nar/gkac1024 (PMC9723621; doi:10.1093/nar/gkac1024)
Supplement: gkac1024_Supplemental_Files [file gkac1024_supplemental_files.zip › ArdK_Fdez21_supplementary_REV.pdf]

Structural basis of direct and inverted DNA sequence repeat recognition by helix-turn-helix transcription factors.

Raul Fernandez-Lopez [a], Raul Ruiz [a], Irene del Campo[a], Lorena Gonzalez-Montes [a], D.Roeland Boer[b], Fernando de la Cruz [a,1] and Gabriel Moncalian[a,1]

[a]Departamento de Biología Molecular, Universidad de Cantabria and Instituto de Biomedicina y Biotecnología de Cantabria (IBBTEC), CSIC-Universidad de Cantabria, 39011 Santander, Spain.

[b]Alba Synchrotron, Cerdanyola del Vallès, 08290 Barcelona, Spain.

Supplementary Data

Tables S1 and S2

Figures S1 to S7

Videos 1 to 6

Table S1. List of oligonucleotides used

Cloning	
IR_DIR	TTGACAAACCGCCCGCATTCCCTGA
AKIR_4	AGTATTGACATTATTTTATTTGC
AKIR_3	TATTTGACATTATTTTATTTGC
AKIR_2	ATTTGACATTATTTTATTTGC
AKIR_1	ATTGACATTATTTTATTTGC
AKIR_0	TTGACATTATTTTATTTGC
AKDR_5	TATAATGTCAATAATTTTATTTG
AKDR_4	TAAATGTCAATAATTTTATTTGC
AKDR_3	TAATGTCAATAATTTTATTTG
Primer extension	
GFP-seq	GGGACAACACCAGTG
Crystallization	
DR_1	GTATTGACACCTATTGACA
DR_2	TGTCAATAGGTGTCAATAC
IR_1	GTAATGTCAATATTTGACA
IR_2	TGTCAAATATTGACATTAC

Table S2. List of plasmids used

Reporter plasmids		
Promoter	Plasmids	Reference
P*ardC*	***pGP4***	14
P*orf7*	***pGP5***	14
P*ssb*	***pGP8***	14
P*orf12*	***pGP10***	14
P*orf14*	***pGP11***	14
P*ir4*	***pIR4***	This work
P*ir3*	***pIR3***	This work
P*ir2*	***pIR2***	This work
P*ir1*	***pIR1***	This work
P*ir0*	***pIR0***	This work
P*dr5*	***pDR5***	This work
P*dr4*	***pDR4***	This work
P*dr3*	***pDR3***	This work
Expression plasmids		
Product	Plasmids	Reference
ArdK	***pAR4***	14
ArdK-H6	***pARA***	This work

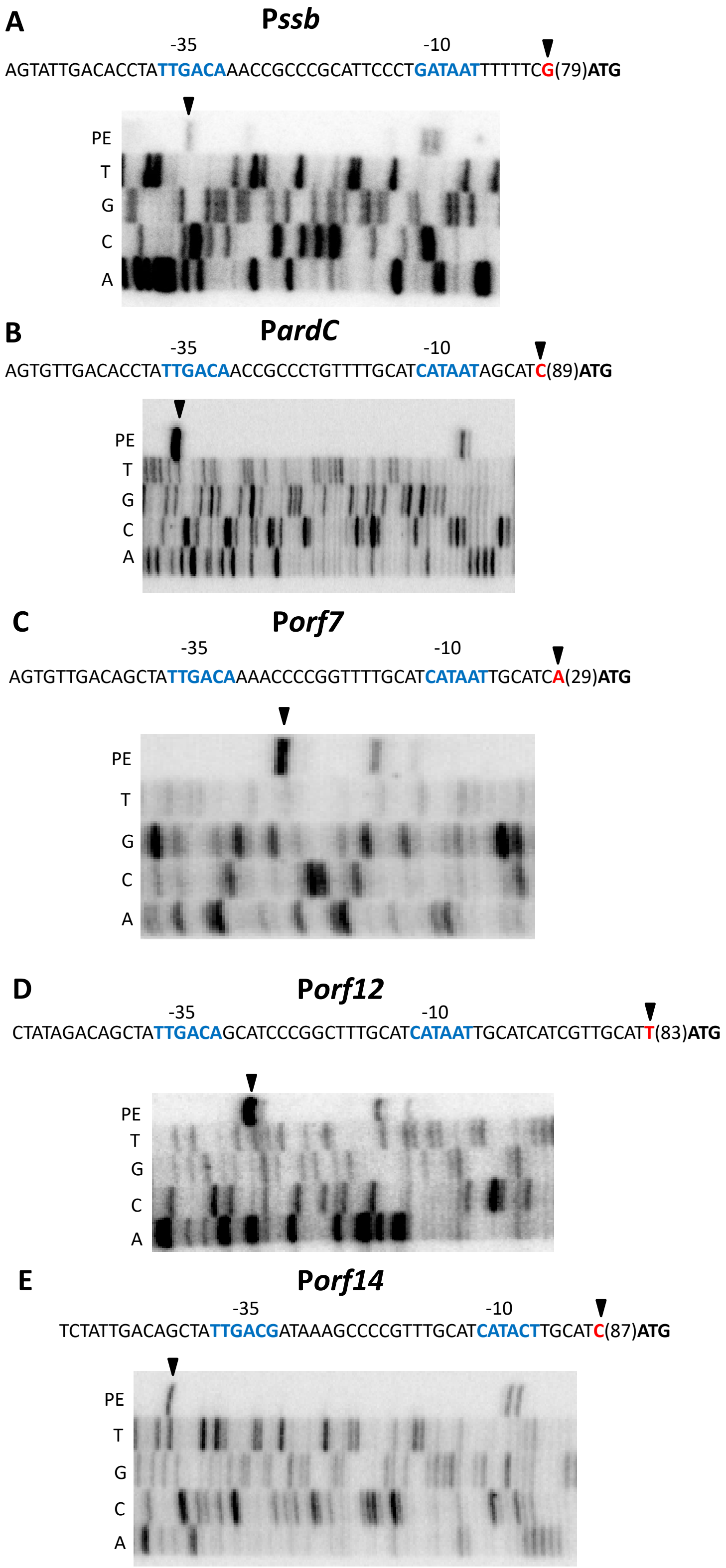

Figure S1. Primer extension mapping of the transcription start sites of the promoters repressed by ArdK. The 17-mer primer oligonucleotide was annealed to total RNA isolated from *E. coli* cells harboring plasmids with the indicated promoters. A,C,G,T sequence ladders were used as DNA size markers. Location of the TSS is shown by a black arrowhead. Migration is rightward, and the sequence on the gel is complementary to the one shown immediately above the image

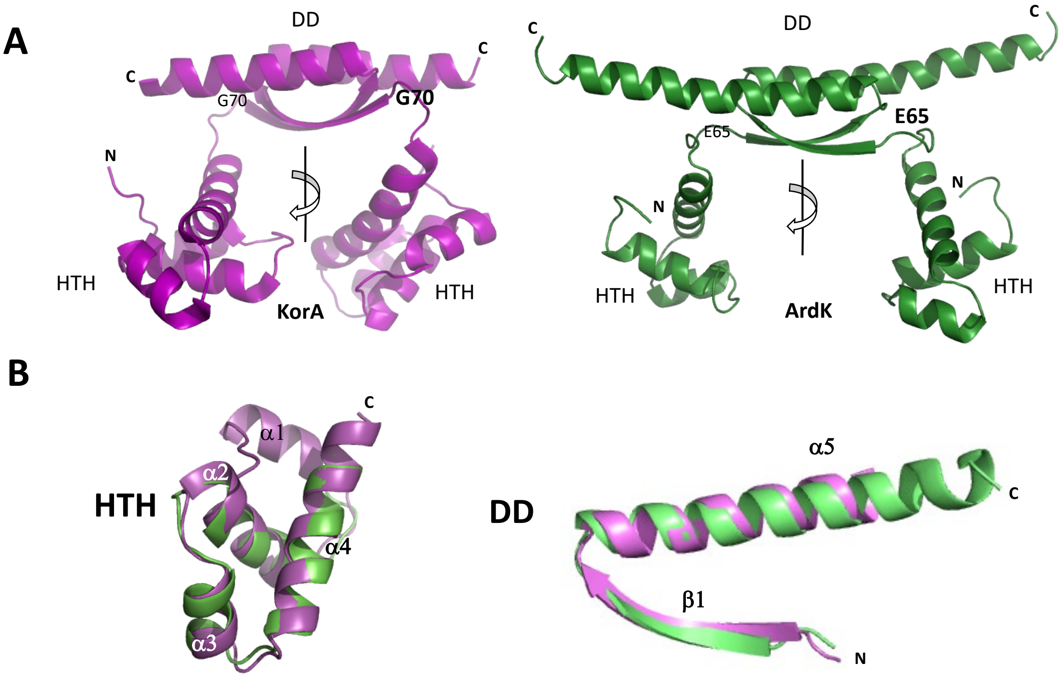

Figure S2. Structural comparison of ArdK_R388 and KorA_RP4. (A)Crystal structures of apo KorA (magenta) and apo ArdK (green) dimers. The location of the HTH and DD domains is shown. The 2-fold symmetry axis within each structure is indicated. Position of residues G70 in KorA and E65 in ArdK is also shown. (B) Structural alignment of ArdK_R388 and KorA_RP4 HTH (left) and DD (right) domains. Secondary structural elements are shown.

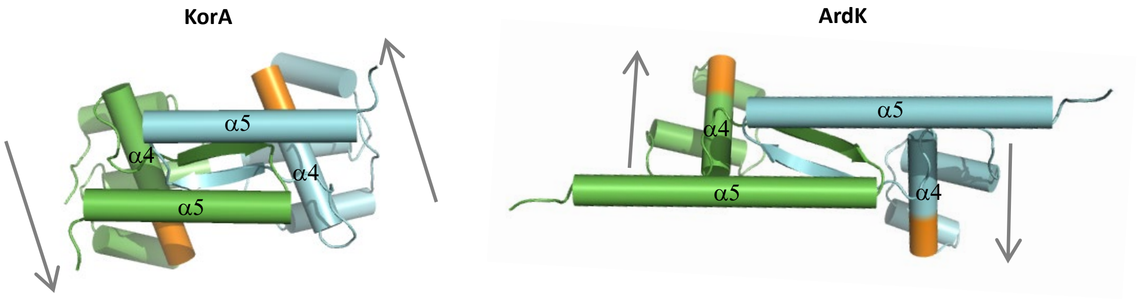

Figure S3. Relative orientation of KorA and ArdK recognition helices. The N-terminal ends of the recognition α4 helices that are inserted into the major groove of the DNA are shown in orange.

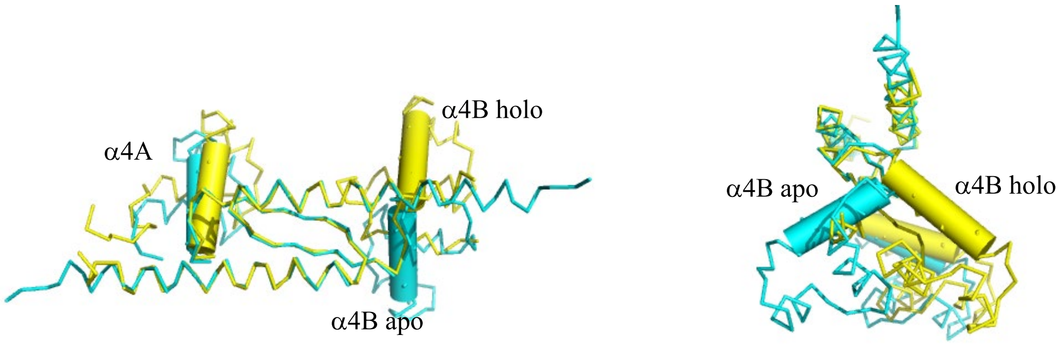

Figure S4. Rotation of the HTH domain in ArdK upon binding to DNA. Two orthogonal views of apo-ArdK (cyan) aligned with holo-ArdK (yellow). DNA is not represented in the structure for clarity. The α4 DNA recognition helices are depicted as cylinders and labelled.

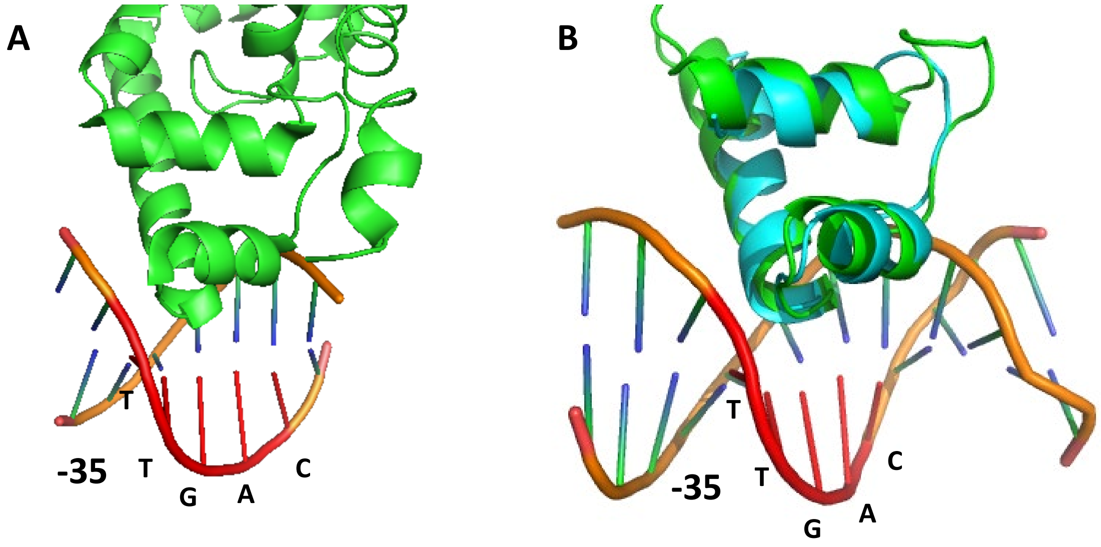

Figure S5. Comparison between ArdK and sigma 70. (A) Structure of *Thermus aquaticus* RNA Polymerase SigmaA Subunit Region 4 Bound to-35 Element DNA (PDB 1KU7). (B) structural aligment of the HTH domain of ArdK and the HTH domain of sigmaA bound to the -35 box.

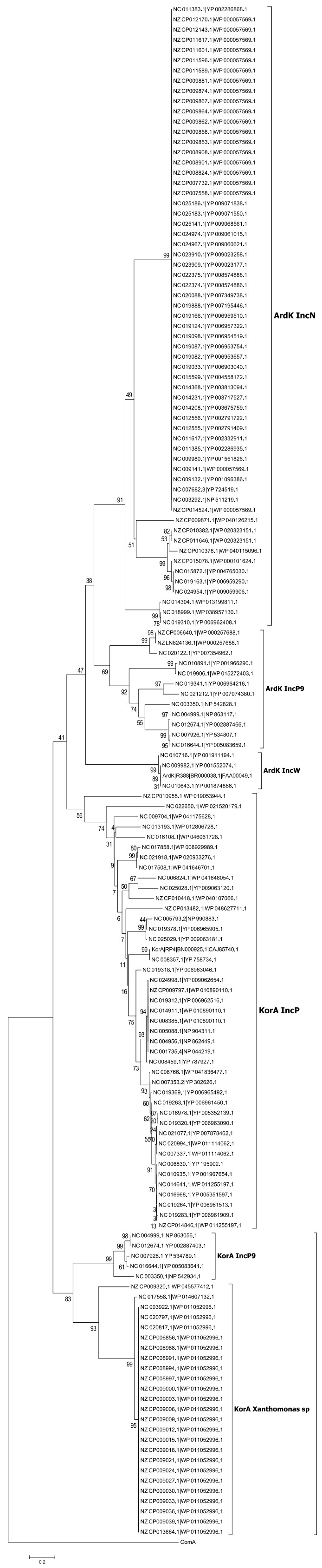

Figure S6. Maximum likelihood phylogenetic tree for KorA/ArdK homologues. Values on the branches indicate the bootstrapping values after 1000 replications. Each protein is indicated by its NCBI accession number.

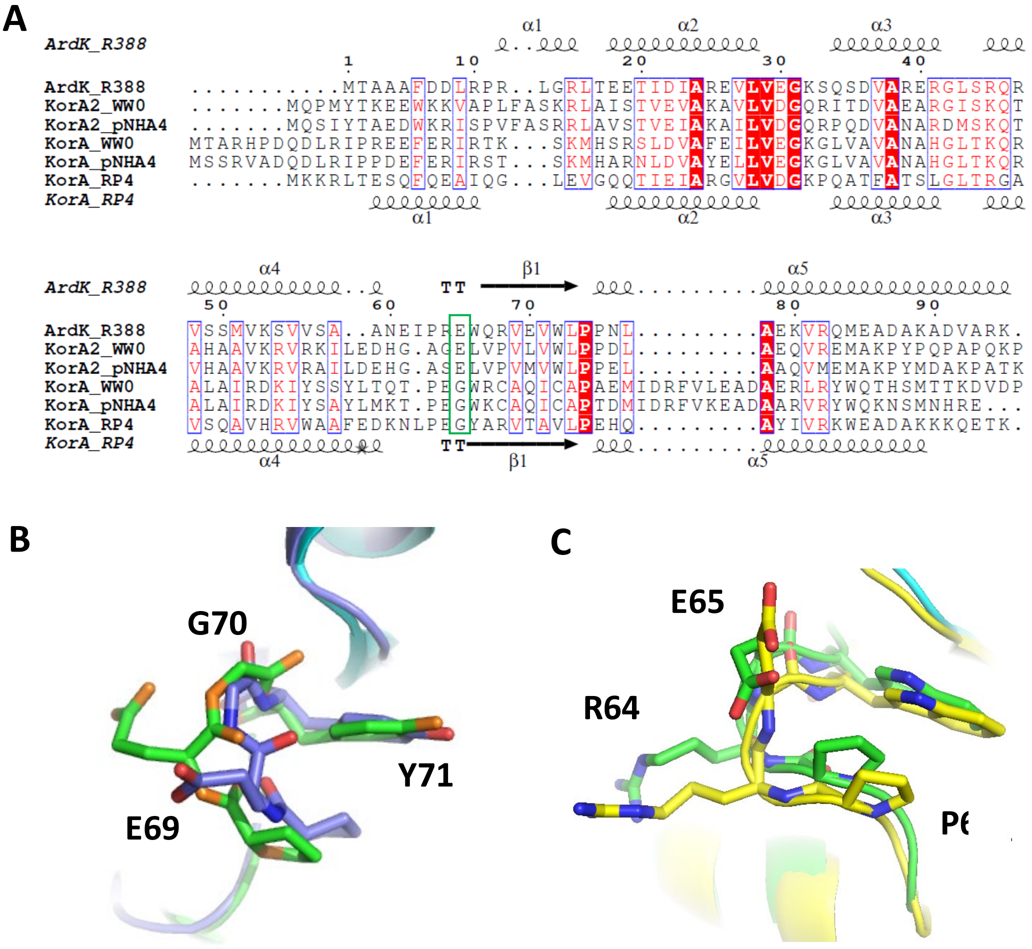

Figure S7. KorA and ArdK hinge regions. (A) Alignment of KorA and ArdK proteins. The location of E65 in ArdK and G70 in KorA is highlighted with a green rectangle (B) Structural alignment of the hinge region of KorA in the apo form (green) and after DNA binding (blue). (C) Structural alignment of the hinge region of ArdK in the apo form (green) and after DNA binding (yellow).

Supplementary videos 1 and 2

Movement of the KorA HTH domains upon DNA binding. The conformational changes were generated and visualized by morphing between apo-KorA and holo-KorA. DNA in the holo-KorA structure is not shown for clarity. Domains are colored as in Fig. 2.

Supplementary videos 3 and 4

Movement of the ArdK HTH domains upon DNA binding. The conformational changes were generated and visualized by morphing between apo-ArdK and holo-ArdK. DNA in the holo-ArdK structure is not shown for clarity. Domains are colored as in Fig. 2.

Supplementary video 5

Movement of the KorA linker region upon DNA binding. The conformational changes were generated and visualized by morphing between apo-KorA and holo-KorA.

Supplementary video 6

Movement of the ArdK linker region upon DNA binding. The conformational changes were generated and visualized by morphing between apo-ArdK and holo-ArdK.
